# Sarcopenia and Physical Frailty: Two Sides of the Same Coin

**DOI:** 10.3389/fnagi.2014.00192

**Published:** 2014-07-28

**Authors:** Matteo Cesari, Francesco Landi, Bruno Vellas, Roberto Bernabei, Emanuele Marzetti

**Affiliations:** ^1^Gérontopôle, Centre Hospitalier Universitaire de Toulouse, Toulouse, France; ^2^INSERM UMR1027, Université de Toulouse III Paul Sabatier, Toulouse, France; ^3^Centro Medicina dell’Invecchiamento, Università Cattolica del Sacro Cuore, Rome, Italy

**Keywords:** elderly, aging, disability, preventive medicine, physical performance, geriatric assessment, skeletal muscle, definition and concepts

Since the last decade, geriatrics and gerontology researchers have been devoting an increasing amount of efforts in the attempt of designing, developing, and implementing preventive interventions against conditions determining/driving the disabling cascade. The urgency of moving ahead in the field is not merely dictated by scientific interests; such need has indeed become a frequent and central item in the agendas of public health authorities (Guralnik et al., 1996). In fact, there is a growing demand for the identification of effective solutions against the detrimental consequences that age-related conditions (in particular, disabilities) exert on our healthcare systems. Special attention has been given to sarcopenia (Janssen et al., 2004) and frailty (Clegg et al., 2013) because both are (1) highly prevalent in the elderly, (2) associated with negative health-related events, (3) potentially reversible, and (4) relatively easy to implement in the clinical practice.

The term “sarcopenia” was coined by Rosenberg to indicate the loss of muscle mass that accompanies aging. He clearly stated that “there is probably no decline in structure and function more dramatic than the decline in lean body mass or muscle mass over the decades of life” (Rosenberg, 1997). The muscle loss was therefore seen as a means of convenience for exploring the aging process and its consequences on an individual’s health. Nevertheless, the skeletal muscle cannot be isolated by the hosting organism. As such, it is still subject to the influence of all the positive and negative stressors to which the organism is exposed. In other words, the endogenous and exogenous phenomena capable of modifying the aging trajectory of the organism can also (more or less directly) influence the quality and quantity of the muscle.

Frailty is the term used to indicate a geriatric syndrome characterized by reduced homeostatic reserves, which exposes the individual at increased risk of negative health-related events (including falls, hospitalizations, worsening disability, institutionalization, and mortality) (Rodríguez-Mañas et al., 2012; Clegg et al., 2013). Different operational definitions have been proposed for capturing the frailty status, each one focusing on specific aspects of the syndrome and detecting slightly different risk profiles (Theou et al., 2014). Nevertheless, there is an overall agreement about the key role that physical function (in particular, mobility) plays in the determination of the status of extreme vulnerability (Ferrucci et al., 2004; Daniels et al., 2008; Abellan van Kan et al., 2009).

Since the beginning (roughly about 15–20 years ago), sarcopenia and frailty have been studied in parallel. Being organ-specific, sarcopenia was more frequently object of research in basic science, whereas the concept of frailty tended to be more easily applied in the clinical setting (Bauer and Sieber, 2008). However, it was quite inevitable that the two would have sooner or later started converging due to their close relationship with the aging process. Unfortunately, the definition of a clear framework within which sarcopenia and frailty can be accommodated and studied has yet to come. One major issue in this context is the long-lasting, tiring, and potentially pointless controversy about the causal relationship existing between the two. Determining whether frailty is due to sarcopenia, or sarcopenia is a clinical manifestation of frailty is consuming considerable efforts, and (from a very practical viewpoint) rather resembles the problem of “the egg and the chicken.”

We realize that the clarification of this point might have major consequences in the field, determining different risk profiles to be detected and, consequently, redrawing outcomes as well as interventions to be adopted. Yet, the isolation of a single pathophysiological determinant responsible for these complex conditions (as well as for any other age-related process) is quite unlikely to be obtained, simply because aging is a complicated and still largely unknown phenomenon (Cesari et al., 2013).

By stating this, we are not surrendering to the current limitations of science. We are instead soliciting the taking of more pragmatic decisions on this topic, waiting that next-to-come scientific advancements allow a better clarification and definition of such urgent and pivotal matters. From this perspective, deconstructing the inner foundations of these “twin” conditions and trying to focus on the shared and clinical relevant features of them might represent a possible solution. By this way, we might have the opportunity to (1) define a unique target for both sarcopenia and frailty, (2) simplify their operational definition, and (3) promote the implementation of the two conditions in both clinical and research settings.

As shown in Figure 1, sarcopenia and frailty are characterized by a unique core condition: the physical function impairment (usually measured by objective tests of gait speed and muscle strength). Such impairment may be responsible for the concurrent existence of a disability as well as represent a consequence of it. It is indeed the presence of disability that influences the framing under which the sarcopenia–frailty relationship should be observed. In fact, in the disabled individual, sarcopenia and frailty might more likely represent the consequences of a permanent disruption of the organism’s homeostasis with limited chances of reversibility. In such situation, sarcopenia rather tends to assume the lineaments of cachexia (Rolland et al., 2011), whereas the frailty status is largely dominated by the disabling condition (Fried et al., 2004). This scenario of tertiary prevention requires the treatment of disability plus ancillary interventions aimed at reducing the risks of complications (Gordis, 2009). The physical function impairment resulting from the combination of sarcopenia and frailty assumes completely different aspects when detected in the absence of disability. In this case, it will represent the first preliminary stage of a process potentially driving the individual toward more severe functional losses and incapacities. In other words, by acting in the preclinical phase of the illness, it will define an ideal target for activities of secondary prevention against disability (Gordis, 2009).

**Figure 1:**
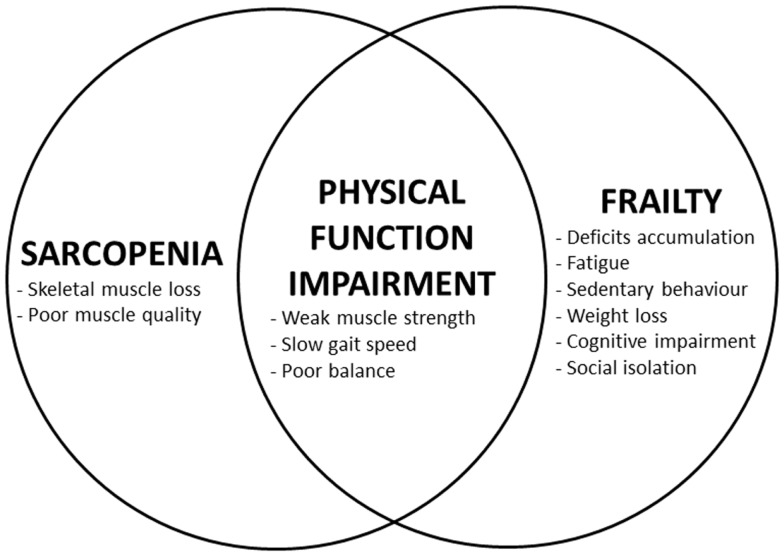
**Relationship among sarcopenia, frailty, and physical function impairment**.

When sarcopenia, frailty, and disability are simultaneously present, disentangling one from the others becomes almost impossible. In fact, the interactions among sarcopenia, frailty, and disability will take the shape of a vicious circle characterized by the exponential and concurrent worsening of all the three. Differently, if disability is absent, the relationship between sarcopenia and frailty might be conceptualized as a vector with a pre-defined direction and for which the only missing information is its sense. Such missing datum is not clinically relevant if the intervention to be put in place is capable of positively influence both the conditions of interest at the same time. To put it differently, by eliminating one condition (i.e., disability) from the framework, the picture becomes clearer and potentially easier to address. Not surprisingly, most of the clinical activities in the field of frailty and sarcopenia are indeed aimed at preventing incident disability (Subra et al., 2012; Maggio et al., 2014).

The shared features that make sarcopenia and frailty particularly appealing in the study of age-related conditions are contended with the common issue represented by their difficult translation from theory into practice. The theoretical definitions of sarcopenia and frailty are both well described and quite unanimously accepted. Nevertheless, both concepts currently lack unique, standardized, and universally agreed operational definitions. Several consensus papers have provided recommendations on how to identify sarcopenic individuals (Cruz-Jentoft et al., 2010; Muscaritoli et al., 2010; Fielding et al., 2011; Morley et al., 2011). Just recently, in order to address the existing inconsistencies, a set of articles by the foundation for the national institutes of health (FNIH) has been published (Alley et al., 2014; Cawthon et al., 2014; Dam et al., 2014; McLean et al., 2014; Studenski et al., 2014). One of the major features of these recent publications (besides of being based on *ad hoc* analyses of large sample populations) is the attempt to better discriminate the specific contributions of the skeletal muscle mass and function in the generation of the negative outcomes (in particular, mobility disability). Similar to sarcopenia, multiple definitions of frailty have also been developed over the last years (Clegg et al., 2013; Theou et al., 2014). Despite the existence of different positions in the scientific community about the concept of frailty and its operationalization, it is noteworthy the publication of a recent report by an international panel of experts (Morley et al., 2013). In the article, the authors (from different schools of thought) unanimously convened about the need of taking a step forward in the study of frailty, avoiding any further delay, and promoting the implementation of the syndrome in clinics and research.

The heterogeneous modalities of measuring sarcopenia and frailty make them difficult to be accepted by public health authorities and regulatory agencies, inevitably endangering advancements in the field. This issue is particularly annoying, especially if it is realized that no assessment tool in medicine will ever be able to accurately replicate the measured condition. In fact, the measurement may be considered as the forcedly limited and arbitrary mean through which we obtain an estimate of a specific phenomenon of the overall health status (mostly unknown to us in its detailed pathophysiological characteristics). The speculative aspect of choosing one operational definition over another is particularly frustrating in geriatrics and gerontology where every condition is watered and confused by the effects of aging at both clinical and subclinical levels (Cesari et al., 2013).

By acknowledging such limitations [which are also responsible for the well known “evidence-based” issue in geriatric medicine (Straus and McAlister, 2000; Scott and Guyatt, 2010)], it becomes reasonable and practical to better pay attention to what really matters in the sarcopenic and/or frail older person. If we isolate the clinical manifestations shared by both sarcopenia and frailty, we might easily agree that physical function is at the very core of the two (Figure 1). In particular, mobility (resulting from the proper functioning of muscles, coordination, and balance) is a capacity common to almost every living being (Dickinson et al., 2000). This implies that animal models focused on mobility may support the development of novel interventions against disability by providing crucial preliminary information (Carter et al., 2012). Mobility decline is a clear manifestation of aging and represents a major negative event of life (Cummings et al., 2014). It is also noteworthy that physical function can easily be measured in an objective way (Studenski et al., 2003), is predictive of adverse outcomes (Guralnik et al., 1994, 1995; Studenski et al., 2011), and represents the clearest (and most obvious) estimate of skeletal muscle production (or in a broader sense, quality) (Lauretani et al., 2003).

Freeing the concepts of sarcopenia and frailty from what can be perceived as only indirectly related to the target organ (i.e., skeletal muscle) may indeed represent a possible solution for combining them into a unique, objective, standardized, and clinically relevant definition (Figure 1). The implementation in clinical and research settings might also be significantly facilitated by the huge body of literature exploring/describing the condition of physical impairment and the validity/acceptance of dedicated instruments [in particular, the short physical performance battery (Guralnik et al., 1994), usual gait speed (Studenski et al., 2011), and handgrip strength (Rantanen et al., 1999)].

In conclusion, we believe there is an urgent need of refining the assessments of sarcopenia and frailty. The physical function impairment occurring in the absence of disability may represent the shared core of the two conditions and optimally serve for (1) defining a novel target for interventions against disability, (2) facilitating the translation of the two conditions in the clinical arena, and (3) providing an objective, standardized, and clinically relevant condition to be adopted by public health and regulatory agencies. Such conceptualization might eventually encourage key stakeholders to join their efforts for more correctly and efficiently approaching the age-related conditions of sarcopenia and frailty, two entities that are still not yet adequately considered.

## Conflict of Interest Statement

The authors declare that the research was conducted in the absence of any commercial or financial relationships that could be construed as a potential conflict of interest.

## References

[B1] Abellan van Kan G. Rolland Y. Andrieu S. Bauer J. Beauchet O. Bonnefoy M. (2009). Gait speed at usual pace as a predictor of adverse outcomes in community-dwelling older people an international academy on nutrition and aging (IANA) task force. J. Nutr. Health Aging 13, 881–889 10.1007/s12603-009-0246-z 19924348 PMC12878092

[B2] Alley D. E. Shardell M. D. Peters K. W. McLean R. R. Dam T. T. Kenny A. M. (2014). Grip strength cutpoints for the identification of clinically relevant weakness. J. Gerontol. A Biol. Sci. Med. Sci. 69, 559–566 10.1093/gerona/glu011 24737558 PMC3991145

[B3] Bauer J. M. Sieber C. C. (2008). Sarcopenia and frailty: a clinician’s controversial point of view. Exp. Gerontol. 43, 674–678 10.1016/j.exger.2008.03.007 18440743

[B4] Carter C. S. Marzetti E. Leeuwenburgh C. Manini T. Foster T. C. Groban L. (2012). Usefulness of preclinical models for assessing the efficacy of late-life interventions for sarcopenia. J. Gerontol. A Biol. Sci. Med. Sci. 67, 17–27 10.1093/gerona/glr042 21636833 PMC3260483

[B5] Cawthon P. M. Peters K. W. Shardell M. D. McLean R. R. Dam T. T. Kenny A. M. (2014). Cutpoints for low appendicular lean mass that identify older adults with clinically significant weakness. J. Gerontol. A Biol. Sci. Med. Sci. 69, 567–575 10.1093/gerona/glu023 24737559 PMC3991141

[B6] Cesari M. Vellas B. Gambassi G. (2013). The stress of aging. Exp. Gerontol. 48, 451–456 10.1016/j.exger.2012.10.004 23103391

[B7] Clegg A. Young J. Iliffe S. Rikkert M. O. Rockwood K. (2013). Frailty in elderly people. Lancet 381, 752–762 10.1016/S0140-6736(12)62167-9 23395245 PMC4098658

[B8] Cruz-Jentoft A. J. Baeyens J. P. Bauer J. M. Boirie Y. Cederholm T. Landi F. (2010). Sarcopenia: European consensus on definition and diagnosis: report of the European working group on sarcopenia in older people. Age Ageing 39, 412–423 10.1093/ageing/afq034 20392703 PMC2886201

[B9] Cummings S. R. Studenski S. Ferrucci L. (2014). A diagnosis of dismobility-giving mobility clinical visibility: a mobility working group recommendation. JAMA 311, 2061–2062 10.1001/jama.2014.3033 24763978 PMC5012417

[B10] Dam T. T. Peters K. W. Fragala M. Cawthon P. M. Harris T. B. McLean R. (2014). An evidence-based comparison of operational criteria for the presence of sarcopenia. J. Gerontol. A Biol. Sci. Med. Sci. 69, 584–590 10.1093/gerona/glu013 24737561 PMC3991139

[B11] Daniels R. van Rossum E. de Witte L. Kempen G. I. van den Heuvel W. (2008). Interventions to prevent disability in frail community-dwelling elderly: a systematic review. BMC Health Serv. Res. 8:278 10.1186/1472-6963-8-278 19115992 PMC2630317

[B12] Dickinson M. H. Farley C. T. Full R. J. Koehl M. A. Kram R. Lehman S. (2000). How animals move: an integrative view. Science 288, 100–106 10.1126/science.288.5463.100 10753108

[B13] Ferrucci L. Guralnik J. M. Studenski S. Fried L. P. Cutler G. B. Jr. Walston J. D. (2004). Designing randomized, controlled trials aimed at preventing or delaying functional decline and disability in frail, older persons: a consensus report. J. Am. Geriatr. Soc. 52, 625–634 10.1111/j.1532-5415.2004.52174.x 15066083

[B14] Fielding R. A. Vellas B. Evans W. J. Bhasin S. Morley J. E. Newman A. B. (2011). Sarcopenia: an undiagnosed condition in older adults. Current consensus definition: prevalence, etiology, and consequences. International working group on sarcopenia. J. Am. Med. Dir. Assoc. 12, 249–256 10.1016/j.jamda.2011.01.003 21527165 PMC3377163

[B15] Fried L. P. Ferrucci L. Darer J. Williamson J. D. Anderson G. (2004). Untangling the concepts of disability, frailty, and comorbidity: implications for improved targeting and care. J. Gerontol. A Biol. Sci. Med. Sci. 59, 255–263 10.1093/gerona/59.3.M255 15031310

[B16] Gordis L. (2009). “The epidemiologic approach to disease and intervention,” in Epidemiology, ed. Gordis L. (Philadelphia, PA: Saunders Elsevier), 1–17

[B17] Guralnik J. M. Ferrucci L. Simonsick E. M. Salive M. E. Wallace R. B. (1995). Lower-extremity function in persons over the age of 70 years as a predictor of subsequent disability. N. Engl. J. Med. 332, 556–561 10.1056/NEJM199503023320902 7838189 PMC9828188

[B18] Guralnik J. M. Fried L. P. Salive M. E. (1996). Disability as a public health outcome in the aging population. Annu. Rev. Public Health 17, 25–46 10.1146/annurev.pu.17.050196.000325 8724214

[B19] Guralnik J. M. Simonsick E. M. Ferrucci L. Glynn R. J. Berkman L. F. Blazer D. G. (1994). A short physical performance battery assessing lower extremity function: association with self-reported disability and prediction of mortality and nursing home admission. J. Gerontol. 49, M85–M94 10.1093/geronj/49.2.M85 8126356

[B20] Janssen I. Shepard D. S. Katzmarzyk P. T. Roubenoff R. (2004). The healthcare costs of sarcopenia in the United States. J. Am. Geriatr. Soc. 52, 80–85 10.1111/j.1532-5415.2004.52014.x 14687319

[B21] Lauretani F. Russo C. R. Bandinelli S. Bartali B. Cavazzini C. Di Iorio A. (2003). Age-associated changes in skeletal muscles and their effect on mobility: an operational diagnosis of sarcopenia. J. Appl. Physiol. 95, 1851–1860 10.1152/japplphysiol.00246.2003 14555665

[B22] Maggio M. Ceda G. P. Lauretani F. (2014). The multidomain mobility lab in older persons: from bench to bedside. Curr. Pharm. Des. 20, 3093–3094 10.2174/138161282019140523115611 24050160

[B23] McLean R. R. Shardell M. D. Alley D. E. Cawthon P. M. Fragala M. S. Harris T. B. (2014). Criteria for clinically relevant weakness and low lean mass and their longitudinal association with incident mobility impairment and mortality: the foundation for the national institutes of health (FNIH) sarcopenia project. J. Gerontol. A Biol. Sci. Med. Sci. 69, 576–583 10.1093/gerona/glu012 24737560 PMC3991140

[B24] Morley J. E. Abbatecola A. M. Argiles J. M. Baracos V. Bauer J. Bhasin S. (2011). Sarcopenia with limited mobility: an international consensus. J. Am. Med. Dir. Assoc. 12, 403–409 10.1016/j.jamda.2011.04.014 21640657 PMC5100674

[B25] Morley J. E. Vellas B. van Kan G. A. Anker S. D. Bauer J. M. Bernabei R. (2013). Frailty consensus: a call to action. J. Am. Med. Dir. Assoc. 14, 392–397 10.1016/j.jamda.2013.03.022 23764209 PMC4084863

[B26] Muscaritoli M. Anker S. D. Argiles J. Aversa Z. Bauer J. M. Biolo G. (2010). Consensus definition of sarcopenia, cachexia and pre-cachexia: joint document elaborated by special interest groups (SIG) “cachexia-anorexia in chronic wasting diseases” and “nutrition in geriatrics”. Clin. Nutr. 29, 154–159 10.1016/j.clnu.2009.12.004 20060626

[B27] Rantanen T. Guralnik J. M. Foley D. Masaki K. Leveille S. Curb J. D. (1999). Midlife hand grip strength as a predictor of old age disability. JAMA 281, 558–560 10.1001/jama.281.6.558 10022113

[B28] Rodríguez-Mañas L. Féart C. Mann G. Viña J. Chatterji S. Chodzko-Zajko W. (2012). Searching for an operational definition of frailty: a Delphi method based consensus statement. The frailty operative definition-consensus conference project. J. Gerontol. A Biol. Sci. Med. Sci. 68, 62–67 10.1093/gerona/gls119 22511289 PMC3598366

[B29] Rolland Y. Abellan van Kan G. Gillette-Guyonnet S. Vellas B. (2011). Cachexia versus sarcopenia. Curr. Opin. Clin. Nutr. Metab. Care 14, 15–21 10.1097/MCO.0b013e328340c2c2 21076295

[B30] Rosenberg I. H. (1997). Sarcopenia: origins and clinical relevance. J. Nutr. 127, 990S–991S 9164280 10.1093/jn/127.5.990S

[B31] Scott I. A. Guyatt G. H. (2010). Cautionary tales in the interpretation of clinical studies involving older persons. Arch. Intern. Med. 170, 587–595 10.1001/archinternmed.2010.18 20386001

[B32] Straus S. E. McAlister F. A. (2000). Evidence-based medicine: a commentary on common criticisms. CMAJ 163, 837–841 11033714 PMC80509

[B33] Studenski S. Perera S. Patel K. Rosano C. Faulkner K. Inzitari M. (2011). Gait speed and survival in older adults. JAMA 305, 50–58 10.1001/jama.2010.1923 21205966 PMC3080184

[B34] Studenski S. Perera S. Wallace D. Chandler J. M. Duncan P. W. Rooney E. (2003). Physical performance measures in the clinical setting. J. Am. Geriatr. Soc. 51, 314–322 10.1046/j.1532-5415.2003.51104.x 12588574

[B35] Studenski S. A. Peters K. W. Alley D. E. Cawthon P. M. McLean R. R. Harris T. B. (2014). The FNIH sarcopenia project: rationale, study description, conference recommendations, and final estimates. J. Gerontol. A Biol. Sci. Med. Sci. 69, 547–558 10.1093/gerona/glu010 24737557 PMC3991146

[B36] Subra J. Gillette-Guyonnet S. Cesari M. Oustric S. Vellas B. (2012). The integration of frailty into clinical practice: preliminary results from the gérontopôle. J. Nutr. Health Aging 16, 714–720 10.1007/s12603-012-0391-7 23076514 PMC12878263

[B37] Theou O. Brothers T. D. Pena F. G. Mitnitski A. Rockwood K. (2014). Identifying common characteristics of frailty across seven scales. J. Am. Geriatr. Soc. 62, 901–906 10.1111/jgs.12773 24697631

